# Drugs Involved in Dyslipidemia and Obesity Treatment: Focus on Adipose Tissue

**DOI:** 10.1155/2018/2637418

**Published:** 2018-01-17

**Authors:** Sofia Dias, Sílvia Paredes, Laura Ribeiro

**Affiliations:** ^1^Department of Biomedicine, Faculty of Medicine, University of Porto, 4200-319 Porto, Portugal; ^2^Department of Endocrinology, Hospital de Braga, 4710-243 Braga, Portugal; ^3^Department of Public Health and Forensic Sciences, and Medical Education, Faculty of Medicine, University of Porto, 4200-319 Porto, Portugal; ^4^I3S-Instituto de Investigação e Inovação em Saúde, University of Porto, 4200-135 Porto, Portugal

## Abstract

Metabolic syndrome can be defined as a state of disturbed metabolic homeostasis characterized by visceral obesity, atherogenic dyslipidemia, arterial hypertension, and insulin resistance. The growing prevalence of metabolic syndrome will certainly contribute to the burden of cardiovascular disease. Obesity and dyslipidemia are main features of metabolic syndrome, and both can present with adipose tissue dysfunction, involved in the pathogenic mechanisms underlying this syndrome. We revised the effects, and underlying mechanisms, of the current approved drugs for dyslipidemia and obesity (fibrates, statins, niacin, resins, ezetimibe, and orlistat; sibutramine; and diethylpropion, phentermine/topiramate, bupropion and naltrexone, and liraglutide) on adipose tissue. Specifically, we explored how these drugs can modulate the complex pathways involved in metabolism, inflammation, atherogenesis, insulin sensitivity, and adipogenesis. The clinical outcomes of adipose tissue modulation by these drugs, as well as differences of major importance for clinical practice between drugs of the same class, were identified. Whether solutions to these issues will be found in further adjustments and combinations between drugs already in use or necessarily in new advances in pharmacology is not known. To better understand the effect of drugs used in dyslipidemia and obesity on adipose tissue not only is challenging for physicians but could also be the next step to tackle cardiovascular disease.

## 1. Introduction

Metabolic syndrome (MS) is a clustering of metabolic abnormalities that increase the risk of developing type 2 diabetes mellitus (T2DM) and cardiovascular disease (CVD). It can be defined as a state of disturbed metabolic homeostasis characterized by aggregation of visceral obesity, atherogenic dyslipidemia, arterial hypertension, and insulin resistance [1]. CVD is the leading cause of mortality worldwide [2], and the growing prevalence of MS will certainly contribute to its burden. Since obesity and dyslipidemia are main features of MS and both can present with adipose tissue (AT) dysfunction, we revised the effects, and underlying mechanisms, of the current approved drugs for both conditions on AT main functions. Our belief is that a thorough understanding of these drug impact on AT is of great clinical value.

## 2. The Adipose Tissue

**Figure 1:**
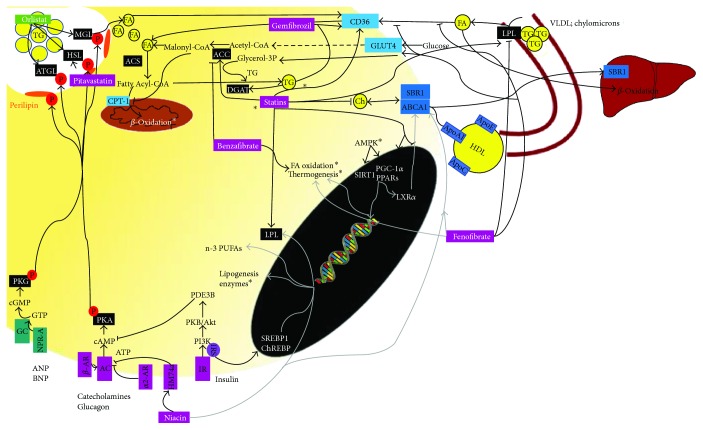
Schematic illustration of the main intracellular pathways and the effects of drugs used in dyslipidemia and obesity in (A) differentiation of preadipocytes into mature adipocytes. This process is on dependence of the PKA pathway, which activates transcriptional factors such as C/EBPB, C/EBPd, C/EBPa, and PPARG that ultimately lead to increase of adipogenesis gene expression. Statins and liraglutide inhibit adipogenesis while niacin and fibrates stimulate, by inducing upregulation of adipogenesis genes (^∗^leptin; adiponectin, FABP4, perilipin, and GLUT4, SCD1) expression and (B) immune and endocrine functions of WAT. Adipocyte exerts autocrine and paracrine actions, through secreting adipokines and also endocrine actions in distant organs. Most of the drugs exhibit an anti-inflammatory role through modulation of adipokine expression. Through modulation of leucocyte chemotaxis, they also affect NK cell activity and macrophage phagocytosis. See text for more details. →: stimulates; ⊣: inhibits; AC: adenylyl cyclase; cAMP: cyclic adenosine monophosphate; PKA: cAMP-dependent protein kinase A; C/EBP: CCATT enhancer-binding proteins; PPARs: peroxisome proliferator-activated receptors; SREBP1: sterol regulatory element-binding protein-1; RXT*α*: retinoid X receptor-*α*; SRE: sterol response elements; SRB1: scavenger receptor 1; NK cells: natural killer cells; CD40: cluster of differentiation 40; CD40L: CD40 ligand; 11B-HSD1: 11B-Hydroxysteroid dehydrogenase type 1; TNF*α*: tumour necrosis factor *α*; IL: interleukin; CCL2 or MCP1: CC-chemokine ligand 2; PAI-1: plasminogen activator inhibitor type 1; AdipoR: adiponectin receptor; IL1RA: IL1 receptor antagonist; IFN-*γ*: interferon-*γ*; TLR: Toll-like receptors; NF*κ*B: nuclear factor kappa B; SRB1: scavenger receptor 1; VCAM1: vascular cell adhesion molecule-1; E-selectin: endothelial-leukocyte adhesion molecule-1; ICAM1: intracellular adhesion molecule-1; OBRb: leptin receptor; ERK: extracellular signal-regulated kinase; MAPK: p38 mitogen-activated protein kinases; iNOS: inducible nitric oxide synthase; ROS: reactive oxygen species; TNFR: TNF receptor; IKKB: NF*κ*B kinase-B; JNK: Jun N-terminal kinase; ER: endoplasmic reticulum; IR: insulin receptor (IR); IRS: insulin receptor substrate; UCP: uncoupling protein; CAP1: adenylyl cyclase-associated protein 1; ET1: endothelin-1.

AT is an active endocrine organ, secreting several hormones called adipokines that act locally and systemically. AT has a major role in several physiological functions, such as in the regulation of food intake and body weight, insulin sensitivity, inflammation, coagulation, or vascular function. AT is populated by different cell types, such as mature adipocytes, preadipocytes, vascular cells, and macrophages [3]. Adipokines and cytokines secreted from these cells influence each other [3] and also a variety of organs. AT also modulates cortisol concentrations through the action of 11B-hydroxysteroid dehydrogenase type 1 (11B-HSD1) that converts cortisone into cortisol [4] (Figure 1). Adiponectin and leptin are the main adipokines produced by adipocytes. Tumour necrosis factor *α* (TNF*α*), interleukin 6 (IL6), IL1, CC-chemokine ligand 2 (CCL2 or MCP1), fractalkine, plasminogen activator inhibitor type 1 (PAI-1), visfatin, and complement factors are also produced by adipocytes, though in lesser extent, and mostly by stromal vascular cells. The main role of adipokines is described below, and the interplay between them is summarized in Figure 1.

*Adiponectin* is the classical anti-inflammatory cytokine, acting through adiponectin receptor (AdipoR) 1/2 to enhance the AMP-activated protein kinase (AMPK) pathway. Adiponectin acts mainly in macrophages, reducing their phagocytic capacity [5], inducing IL10 and IL1 receptor antagonist (IL1RA) production [5], suppressing interferon *γ* (IFN*γ*) production [5], and inhibiting the activation of Toll-like receptor- (TLR-) induced nuclear factor kappa B (NF*κ*B) pathway [6]. Although the differences between low- (LMW) and high- (HMW) molecular weight adiponectins are not completely clarified, both forms induce activation of the AMPK pathway and suppression of scavenger receptor class B type 1 (SRB1) expression by macrophages [7]. Nevertheless, only the LMW form is responsible for inducing IL10 and for suppressing IL6, through peroxisome proliferator-activated receptor (PPAR) stimulation [7]. In contrast, the HMW adiponectin can induce CXC-chemokine ligand 8 (CXCL8; also known as IL8) expression in response to an inflammatory stimulation [7]. In endothelial cells, adiponectin modulates the inflammatory atherosclerosis process, by inhibiting the expression of adhesion molecule vascular cell adhesion molecule-1 (VCAM1), endothelial leukocyte adhesion molecule-1 (E-selectin), and intracellular adhesion molecule-1 (ICAM1) induced by TNF*α* [8]. Moreover, it can induce B-oxidation in the liver while decreasing the expression of sterol regulatory element-binding protein 1 (SREBP1) therefore inhibiting lipogenesis. *Leptin* is a proinflammatory cytokine that acts through the leptin receptor (OBRb), activating the cyclic adenosine monophosphate- (cAMP-) dependent protein kinase A (PKA) extracellular signal-regulated kinase (ERK) 1/2 and p38 mitogen-activated protein kinase (MAPK) pathways [9]. Through the activation of these intracellular signaling pathways, leptin upregulates the expression of TNF*α*, IL6, and IL12 in macrophages [10]. It also has a role in controlling appetite, angiogenesis, haematopoiesis, the neuroendocrine system, and immunity [11]. Indeed, leptin can regulate neutrophil chemotaxis and natural killer cell (NK cell) activity [12]. It also upregulates inducible nitric oxide synthase (iNOS) expression in AT thereby increasing the production of reactive oxygen species (ROS), mostly from macrophages [13, 14]. By this mechanism, leptin induces macrophage phagocytosis and differentiation of monocytes. TNF*α* activates TNF receptor (TNFR) which activates the inhibitor of NF*κ*B kinase-B (IKKB) that in turn stimulates the NF*κ*B pathway [15]. In addition, TNF (and also TLR stimulation) can also stimulate the JUN N-terminal kinase (JNK) family of serine/threonine protein kinases [16], a mechanism that promotes insulin resistance [17], and also decrease of insulin sensitivity since endoplasmic reticulum (ER) stress leads to insulin receptor substrate 1 (IRS1) phosphorylation [18]. TNF*α* increases the expression of iNOS in adipocytes, which appears to suppress uncoupling protein (UCP) 2 expression decreasing white AT (WAT) energy expenditure [19]. *Resistin*, a proinflammatory cytokine, whose production is enhanced by other proinflammatory cytokines [20], acts through the activation of adenylyl cyclase-associated protein 1 (CAP1) in monocytes, which increases cAMP concentration, PKA activity, and NF*κ*B, therefore increasing the expression of IL1, IL6, TNF*α*, and IL12 upon different types of cells [20, 21]. Moreover, resistin is able to induce the expression of VCAM1, ICAM1, and CCL2 in endothelial cells, inducing endothelin-1 (ET1) secretion [22]. This mechanism might explain resistin contribution to atherosclerosis. *MCP1* is a potent chemoattractant of both monocytes and macrophages to AT that acts through the CCL2 receptor (CCR2) [23]. *Fractalkine* (or CX3CL1) and its receptor (CX3CR1) are also involved in this process [24]. *PAI-1* is a prothrombotic agent, inhibitor of plasminogen activators, whose expression is induced by TNF*α* and oxidative stress [25], insulin, glucocorticoids, angiotensin II, fatty acids (FA), TNF*α*, and TGFB [26, 27]. It negatively affects metabolism and local vascular biology by interacting with the renin-angiotensin-aldosterone system. Moreover, PAI-1 suppresses adipocyte differentiation in adipocytes [28]. *Visfatin* acts as a proinflammatory cytokine [29] through binding to the insulin receptor, though at a different site than insulin [30]. It also induces adipocyte differentiation [30].

AT is the main regulator of the whole body fat storage. Lipid deposition and mobilization are complex metabolic pathways highly modulated and affected by several hormones. Lipid mobilization is enhanced in fasting conditions. Likewise, glucagon, catecholamines (through B-adrenoceptors (AR)), and atrial or brain natriuretic peptide (ANP/BNP) promote lipolysis [31] releasing glycerol and FA. Our results showing that FA affect catecholamine handling by chromaffin cells suggest not only that these amines are mediators in the well-known relationship between unsaturated FA, MS, and CVD but also that a releasing vicious cycle can aggravate and perpetuate these conditions [32]. In lipolysis, after hormonal stimulation, there is an activation of adenylate cyclase (AC), which triggers the cAMP-PKA pathway and consequently phosphorylation of lipases [33]. Natriuretic peptides (NPs) trigger a distinct intracellular path as they act trough the cyclic guanosine monophosphate- (cGMP-) dependent protein kinase (PKG) pathway, by activating NPR-A-dependent guanylyl cyclase (GC) [34]. These pathways activate adipocyte triglyceride (TG) lipase (ATGL), hormone-sensitive lipase (HSL), and monoacylglycerol lipase (MGL) [35], and their sequential action leads to the hydrolysis of TG into diglycerides and ultimately into monoglycerides. Also, phosphorylation of perilipin, a lipid droplet-associated protein, causes its decoupling from lipid droplets, which promotes lipolysis by allowing HSL to gain access to it [36–38]. This process culminates with the release of FA and glycerol and their uptake by other tissues (FA mainly used by the skeletal muscle, liver, and heart in energy production and glycerol by the liver in gluconeogenesis). In contrast, catecholamines, through a2AR, and insulin inhibit lipid mobilization [31]. Insulin inhibits this process through the phosphoinositide 3-kinase-dependent (PI3K) pathway, protein kinase B (PKB/Akt), and activation of phosphodiesterase 3B (PDE3B), which degradates cAMP. By lowering cAMP levels and by inhibiting adenylyl cyclase (AC) through an inhibitory GTP-binding protein- (Gi-) coupled receptor, insulin inhibits the PKA pathway and ultimately lipolysis [39].

FA B-oxidation is responsible for mitochondrial breakdown of long-chain acyl-CoA to acetyl-CoA used for mitochondrial energy production. The PPARs and PPAR*γ* coactivator 1 (PGC1*α*) are the most well-known transcriptional regulators of FA B-oxidation [40]. Both regulators enhance the expression of proteins involved in this process such as acyl-CoA synthetase (ACS), fatty acid translocase (CD36/FAT), malonyl-CoA decarboxylase (MCD), and carnitine palmitoyl transferase 1 (CPT1). In this context, FA can undergo the action of ACS and CPT1 and thereby be used to B-oxidation. AMPK phosphorylates PGC leading to its activation, and on the contrary, sirtuin 1 (SIRT1) (a protein deacetylase involved in stress cellular regulation) deacetylates PGC-1*α*. PGC-1*α* induces the expression of PPAR*α*, mostly expressed in highly metabolic tissues such as the liver, heart, skeletal muscle, and brown AT (BAT), enhancing the expression of mitochondrial and B-oxidation genes [40].

**Figure 2:**
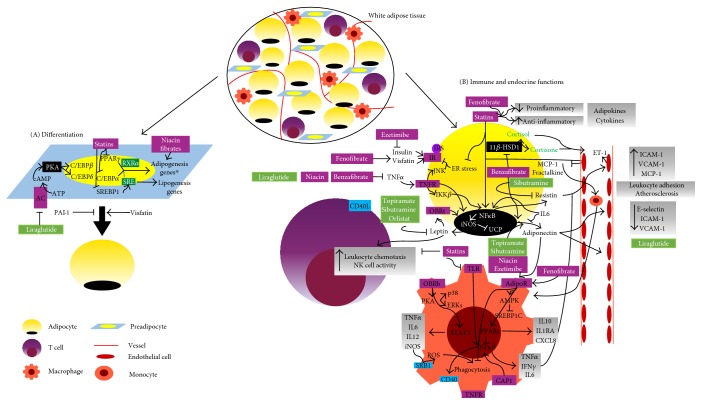
Schematic illustration of the main intracellular pathways underlying WAT metabolic functions and the effects of drugs used in dyslipidemia and obesity on these pathways: *B-oxidation* (^∗^through upregulation of ACS, CD36, MCD, and CPT1 genes and after stimulus as PPAR*α* agonist or adrenergic agonists, through upregulation of the AMPK pathway); *lipolysis* (sequentially by ATGL, HSL, and MGL actions); *lipogenesis* (^∗^through upregulation of GLUT4; ACC genes). Statins induce adipocyte FA uptake, increasing LPL expression, while decreasing cholesterol release. ^∗^Only in obese models, statins stimulate lipogenesis de novo; globally, fibrates inhibit lipogenesis and stimulate FA oxidation and thermogenesis (^∗^through upregulation of PRDM16, PPAR-*γ*, and UCP-1 gene expression); niacin inhibits lipolysis and increases lipogenesis gene expression; orlistat enhances TG degradation. See text for more details. →: stimulates; ⊣: inhibits; TG: triglycerides; FA: fatty acid; Ch: cholesterol; LPL: lipoprotein lipase; VLDL: very low-density lipoproteins; DGAT: diacylglycerol acyltransferase; ACC: enzyme acetyl-coenzyme A carboxylase; SREBP1: sterol regulatory element-binding protein 1; ChREBP: carbohydrate response element-binding protein; GLUT4: glucose transporter type 4; PI3-K: phosphoinositide 3-kinase dependent; PDE3B: phosphodiesterase 3B; AC: adenylyl cyclase; cAMP: cyclic adenosine monophosphate; PKA: cAMP-dependent protein kinase A; PKB/Akt: protein kinase B; GC activity: guanylyl cyclase activity; cGMP: cyclic guanosine monophosphate; PKG: cGMP-dependent protein kinase; ATGL: adipocyte triglyceride lipase; HSL: hormone-sensitive lipase; MGL: monoacylglycerol lipase; PPARs: peroxisome proliferator-activated receptors; PGC-1a: peroxisome proliferator-activated receptor G coactivator 1; ACS: acyl-CoA synthetase; CD36/FAT: fatty acid translocase; CPT1: carnitine palmitoyl transferase 1; AR: adrenoceptors; ANP/BNP: atrial or brain natriuretic peptide; NPR-A: natriuretic receptor A; IR: insulin receptor; IRS: insulin receptor substrate; AMPK: adenosine monophosphate-activated protein kinase; SIRT1: sirtuin 1; BAT: brown adipose tissue; HDLs: high-density lipoproteins; LXR*α*: liver X receptor alpha; ABCA1 transporter: ATP-binding cassette A1 transporter; SRB1: scavenger-receptor 1.

In lipid mobilization, high-density lipoproteins (HDLs) are responsible for reversing cholesterol transport, transporting cholesterol from extrahepatic tissues (including arterial macrophages and AT) to the liver [41]. These lipoproteins are composed of cholesterol, triglycerides, phospholipids, and apolipoprotein A (mainly apoA-I and apoA-II), apo-C, and apo-E. The interaction between apoA-I and surface receptors in peripheral tissues, namely, ATP binding cassette A1 (ABCA1) transporters and SRB1, is responsible for cholesterol transportation [41], and once filled, HDL delivers cholesterol into the liver [41]. Lipid deposition can occur by uptake of circulating FA (in a higher extent) and lipogenesis de novo from nonlipid precursors. The former is driven by lipoprotein lipase (LPL), secreted by adipocytes, and located in the capillary lumen [42]. This enzyme catalyzes TG hydrolysis associated to lipoproteins (such as very low-density lipoprotein (VLDL) or chylomicrons) into FA, facilitating their uptake by adipocytes [43]. Upon uptake, FA can suffer re-esterification with glycerol-3-phosphate (glycerol-3P) leading to TG synthesis [44]. This process is catalyzed by diacylglycerol acyltransferase (DGAT) and stimulated by insulin [44]. Through lipogenesis de novo, insulin induces glucose uptake by adipocytes (via glucose transporter type 4 (GLUT4)) that is then converted to acetyl-coenzyme A through glycolysis. Acetyl-CoA is converted by acetyl-coA carboxylase (ACC) to malonyl-CoA, leading to FA synthesis [45]. At the same time, insulin inhibits FA translocation to mitochondria and therefore B-oxidation [46]. The metabolic functions of AT are summarized in Figure 2.

Adipogenesis (summarized in Figure 1) is a tightly regulated cellular differentiation process through which preadipocytes are converted into mature adipocytes. It is essential to the renewing of AT and modulation of fat depots. Adipogenesis comprises two phases: (1) the commitment of pluripotent stem cell to a unipotent preadipocyte and (2) differentiation of preadipocytes into mature adipocytes (Figure 1). In the first phase, bone morphogenetic proteins (BMPs) and TAK1 pathways are involved, whereas in the second terminal differentiation phase, other transcription factors such as PPAR*γ*, CCATT enhancer-binding proteins (C/EBP), and SREBP1 take action [47]. After hormonal stimulation, there is an increase in intracellular cAMP, leading to transcription of C/EBPB and C/EBPD in preadipocytes, which translocate to the nucleus and enhance the expression of C/EBPa and PPAR*γ* [48]. PPAR*γ* heterodimerizes with retinoid X receptor-*α* (RXR*α*) and binds to DNA, promoting transcription of the adipocyte-specific genes, leptin, adiponectin, fatty acid-binding protein-4 (FABP4), and perilipin [49]. In addition, C/EBPa also enhances the transcription of leptin and FABP4, as well as other genes, such as GLUT4 and stearoyl-CoA desaturase-1 (SCD1) [50]. During differentiation, SREBP1 is activated and translocated into the nucleus, where it binds to sterol response elements (SRE) and induces the expression of lipogenic enzymes such as ACC, fatty acid synthase (FAS), LPL, and SCD1 [47, 51].

## 3. Obesity and Dyslipidemia: Two Disorders Walking Together

Obesity and dyslipidemia are two main features of MS. Dyslipidemia refers to a range of lipid profile disorders, resulting from quantitative (higher or lower lipid and/or lipoprotein levels) or qualitative modifications (structural lipoprotein changes) [52] and is a primary major risk factor for CVD [53]. Obesity is a multifactorial disease, characterized by a local and systemic chronic low-grade inflammatory state causing metabolic abnormalities and adipocyte dysfunction [54], that ultimately leads to CVD. This state has also been implicated in the development of obesity-related comorbidities [55], and the growing MS prevalence seems to be closely related to the obesity epidemic [56]. Additionally, obesity also seems to be associated with the rising prevalence of dyslipidemia, as several studies have suggested a positive correlation between body mass index (BMI) and dyslipidemia [57, 58]. Moreover, when obesity is concomitant with AT dysfunction, ectopic fat accumulation, especially in liver, and inflammation, it favours the development of dyslipidemia [59].

## 4. Drugs Used in Dyslipidemia

### 4.1. HMG-CoA Reductase Inhibitors (Statins)

3-Hydroxy-3-methyl-glutaryl-coenzyme A (HMG-CoA) reductase inhibitors, also known as statins, act by inhibiting in a competitive manner the conversion of HMG-CoA to mevalonic acid. Statins increase the expression of LDL receptors in the liver, increasing LDL catabolism and lowering total cholesterol causing a reduction of 21–55% and 6%–30%, respectively, of LDL and TG and an increase of 2%–10% in HDL. New-onset diabetes may be increased in patients treated with statins, though seeming to be dose related and less common with pravastatin and possibly pitavastatin [53].

#### 4.1.1. Effects on Adipocyte Metabolic Functions

AT acts as buffer of plasmatic cholesterol, and statins have an important role decreasing basal cholesterol release [60] and content [61] in adipocytes. In fact, statins are capable of reverting basal cholesterol release from adipocytes, though not after apoA1 stimulation (inducing cholesterol release and apoE secretion by adipocytes) [60]. In mature adipocytes, pitavastatin upregulates HSL expression, enhancing lipolysis and decreasing lipid accumulation, preventing adipocyte hypertrophy, and increasing the number of small adipocytes [62]. Intensive treatment with atorvastatin also leads to the regression of epicardial AT volume [63]. Statins seem to increase mRNA LPL expression in preadipocytes [64, 65] as well as LPL activity in 3T3-L1 preadipocytes [64] and adipocytes [65]. Different transcription factors, such as SREBP and PPAR*γ* [64], are involved, and these effects contribute to lower TG and VLDL levels [64, 65]. In contrast, it has been shown that in a bone marrow stromal cell model statin can reduce LPL mRNA expression [66].

#### 4.1.2. Effects on Inflammation

Many studies propose an anti-inflammatory role for statins in AT. In response to a stressful and inflammatory stimulus, there is an increase of proinflammatory adipokine and cytokine expression, such as leptin [67–71], resistin [67, 72, 73], IL-6 [67, 74–78], PAI-1 [79–81], MCP-1 [77, 78, 80, 82, 83], visfatin [71], and TNF*α* [67, 68, 71, 77, 82, 83]. Statins can reduce the expression of these cytokines and adipokines [62, 67–77, 79–82], while enhancing anti-inflammatory adipokine expression and secretion by adipocytes [62, 67, 68, 73, 76, 80]. For instance, by upregulating PPAR*γ* expression in adipocytes, statins decrease IL6 expression and plasma concentration [74]. Statins have shown to reduce proinflammatory cytokines, such as high-sensitivity C-reactive protein (CRP) plasma levels [68]. They inhibit leptin expression due to RNA-processing changes that reduce heterogeneous nuclear RNA abundance [69] and also act by decreasing IL-6 [70]. Statins can also contribute to inhibit ER stress [82] by decreasing cholesterol levels. Moreover, the combination of statins and fibrates [67] or the combination of ezetimibe and simvastatin causes even a greater effect in reducing proinflammatory adipokines and increasing adiponectin levels [71]. Statins inhibit the PI3K pathway through the suppression of protein prenylation, activating PKA and consequently suppressing leptin expression in 3T3-L1 cells. The reduced mRNA C/EBPa expression seems to be partially involved in this last effect, emphasizing the importance of leptin in adipocyte differentiation [69]. Statins reduce the expression of resistin in human monocyte/macrophages in vitro and in 3T3-L1 adipocytes [73, 82]; however, these results were not confirmed in in vivo studies after 6 months of atorvastatin treatment [82].

Statins appear to inhibit PAI-1 promoter activity, through mitogen-activated protein kinase kinase kinase 1 (MEKK1) and, in a lesser extent, NF*κ*B. Since isoprenoids, such as geranylgeranyl pyrophosphate and farnesyl pyrophosphate, are able to revert rosuvastatin effect on PAI-1 expression, protein geranylation or/and farnesylation could be one involved mechanism [69].

In animal studies, statins are able to decrease inflammation in pericarotidal AT from high-fat diet- (HFD-) treated mice [77] and in WAT of hypercholesterolemic pigs [84]. This effect is achieved by downregulation of 5lipoxygenase, decrease of macrophagic infiltration [77], and downregulation of proinflammatory adipokines/cytokines [77]. In hypercholesterolemic pigs, statins also prevent WAT adipocyte hypertrophy and diminish T lymphocyte infiltration [84]. Statins can partially refrain AT inflammation in obese mice [78], through the downregulation of mRNA MCP1 and IL6 expression [78] and also through inhibition of TLR4-induced expression of interferon-*γ* in macrophages [78].

Statins have distinct roles in the regulation of iNOS according to the cell type. In 3T3-L1 preadipocytes, these drugs inhibit NO production in response to inflammatory stimuli, via the decrease of iNOS mRNA expression mediated by NF*κ*B pathway inhibition [75]. In contrast, in 3T3-L1 mature adipocytes, statins enhance iNOS expression and NO levels [85]. This effect is dependent on the type of statin [85], and NF*κ*B activation seems to be the underlying mechanism, which contributes to upregulation of the iNOS gene and ultimately to NO production [85]. Moreover, NF*κ*B activation is also achieved through diminishing the metabolites of cholesterol synthesis, such as isoprenoid and small G proteins [85].

#### 4.1.3. Effects on Atherogenesis

Atherogenesis is a degenerative process in which artery walls become occupied with excessive and modified lipids from circulation [86]. The ingestion of LDL and modified or oxidized LDL (oxLDL) by macrophages causes accumulation of cholesterol esters and formation of “foam cells” leading to atherogenesis. Moreover, vascular smooth muscle cells migrate from the media into the intima and proliferate, giving rise to atherosclerotic plaques [87]. During the process of atherogenesis, macrophage phagocytosis of oxLDL is mediated by SRB1 [86]. Adipocytes can also uptake oxLDL, a mechanism positively correlated with PPAR*γ* and SRB1 expression and negatively with LDL levels [88, 89]. Statins are able to induce PPAR*γ* and SRB1 expression in adipocytes [88, 90], suggesting both indirect and direct effects, respectively, through lowering cholesterol and SRB1 stimulation [90]. Moreover, as statins reduce lipid accumulation in adipocytes [62], there is a disinhibition of PPAR*γ* expression per se potentially enhancing the oxLDL uptake by adipocytes [90].

By decreasing the expression of proinflammatory adipokines and increasing the anti-inflammatory ones, statins have a fundamental role in inflammatory-related processes like atherosclerosis [68, 73].

#### 4.1.4. Effects on Insulin Sensitivity

Recently, evidence on the association between insulin resistance, T2DM de novo, and statin treatment has been increasing. Caveolae are plasma membrane microdomains, composed by cholesterol, sphingolipids, and different coat proteins named caveolins, considered anchor points to molecules (in this context, insulin receptor and GLUT4), facilitating their interaction, in order to activate cell signaling and transport [91–93].

Caveolins are modulated by cavins, and cavin2 is pointed out as a cholesterol-dependent protein essential to define caveolar structure [89]. Through cholesterol depletion, statins cause caveolae collapse in adipocytes inducing proteasomal degradation of cavin2 and redistribution of cavin-1 to the cytosol [89]. Taking into account the importance of caveolae in insulin signaling [91, 92], insulin resistance can result, at least partially, from caveolae dysfunction. Moreover, statin disruption of caveolar formation seems to reduce HMW adiponectin secretion by adipocytes [61], a mechanism that reduces insulin sensitivity.

Lipophilic statins can also induce GLUT structural alterations [94] and impair GLUT4 protein expression [95] inhibiting GLUT4 translocation and consequently decreasing insulin-stimulated glucose uptake in 3T3-L1 adipocytes [96]. Although lipophilic, atorvastatin can improve insulin sensitivity in an obese mice model [97], through an increase in mRNA and protein expression of the slc2a4 gene (which codifies GLUT4) and a decrease in mRNA and protein expression of IL6 in subcutaneous AT (SCAT) [97]. The same authors suggest the involvement of the IKK/NF*κ*B pathway in these effects [97]. On the other hand, hydrophilic statins generally improve insulin sensitivity, even in HFD-induced overweight mice, with no changes in body weight, AT mass, and adipocyte size [98, 99]. These statins also increase PPAR*γ* and GLUT4 expression and reduce leptin expression in AT [98, 99]. Hydrophilic statins augment basal and insulin-stimulated glucose uptake in AT [83, 100], thus improving hyperglycemia.

#### 4.1.5. Effects on Adipogenesis

Statins inhibit preadipocyte differentiation through downregulation of PPAR*γ* 2 and 422aP. Instead, they induce upregulation of RunX2/Cfbal, promoting osteoblastic differentiation [101]. It has been shown that statins stimulate osteoblastic differentiation, proliferation, maturation, and synthesis of the new bone [66, 101, 102]. Statins also inhibit adipogenesis through the reduction of LPL mRNA expression [66]. In a 3T3-F442A cell model, statins markedly inhibit adipocyte differentiation comparing to 3T3-L1 cells [103].

An exception is pitavastatin that does not affect preadipocyte differentiation/maturation *in vitro* [62]. On the other hand, mevastatin inhibits orbital preadipocyte differentiation through blockage of PPAR*γ* expression [104]. In the early phase of adipogenesis, statins seem to induce cellular phenotypic changes leading to 3T3-L1 cell rounding-up and detachment [105], an absent effect in the late phase of adipogenesis [105]. Statins downregulate the expression of crucial genes for adipocyte differentiation including C/EBPa, PPAR*γ*, SREBP1, and maturation markers such as leptin, FABP4, and adiponectin [103]. The inhibition of isoprenoid synthesis and the PI3K and Ras-Raf1-MAPK pathways are possible mechanisms behind this effect [105]. More recently, it has also been proposed that statins, by reducing mevalonate-derived nonsterol isoprenoids (intermediate metabolites of cholesterol biosynthesis, crucial to adipocyte differentiation [106]), can cause compensatory upregulation of HMG-CoA reductase [103]. On the other hand, *in vivo* studies have shown that statins stimulate adipocyte differentiation [107, 108], contradictory results emphasizing the complex mechanisms underlying AT differentiation.

### 4.2. Fibric Acid Derivatives (Fibrates)

Fibrates decrease plasma TG-rich lipoproteins [109]. These drugs are able to increase lipoprotein lipolysis, but they also increase FA hepatic uptake and reduce hepatic TG production [110], achieving TG reductions of 35 to 50%. Fibrates increase HDL cholesterol by 5–20%, owing to an increase in apoA1 and apoA2 production in the liver, which may contribute to a more efficient reverse cholesterol transport [110], and also to activation of PPAR*α* [111]. LDL cholesterol generally decreases in individuals with elevated baseline plasma concentrations [109] with reductions of 20–25% [53]. Fibrates convert small cholesterol-depleted LDL particles to large-cholesterol-enriched LDL particles, more efficiently removed from circulation [110] thus improving atherogenic profile [53]. Fibrates are synthetic ligands for PPAR*α* [112] that by stimulating the peroxisome proliferator response element (PPRE) increase FA hepatic B-oxidation, reduce TG hepatic secretion, and increase LPL activity and subsequently VLDL clearance [111]. However, the PPAR*α* agonist gemfibrozil may increase LDL levels by 10%–15% [53].

#### 4.2.1. Effects on Adipocyte Metabolic Functions

Bezafibrate, a nonselective PPAR (A, D/B, and G) agonist, regulates energy homeostasis by the upregulation of PPAR*α* and UCP-1, 2, and 3 [113, 114]. It stimulates FA oxidation in adipose mitochondria and peroxisomes by increasing the mRNA acyl-CoA oxidase (ACO) expression [113–115]. The oxidative rate is higher in preadipocytes than in mature adipocytes [113]. Moreover, the decrease in FA levels leads to the inhibition of lipogenesis [113]. On the contrary, gemfibrozil induces a fast increase in TG synthesis in both preadipocytes and adipocytes [116]. Indeed, gemfibrozil improves cellular capacity for substrate uptake (glucose and oleate) and enhances the activity of the enzymes needed for this synthesis [116]. By increasing FA uptake and TG synthesis in peripheral tissues, gemfibrozil decreases FA plasma levels, which in turn enhances extracellular hydrolysis by LPL present on endothelial cells [116].

Fenofibrate, another PPAR*α* agonist, is able to decrease body mass, independently of food intake [117], and to reduce visceral AT (VAT) mass [103] through PPAR*α* stimulation and upregulation of FA oxidation enzymes in AT, such as CPT1 [118, 119] and ACO [119]. This drug also increases the number of small adipocytes to the detriment of large ones in diet-induced obese and insulin-resistant mice [103]. Fenofibrate can increase energy expenditure in diet-induced obese mice [117] owing to its ability to upregulate via PPAR*α* pathway thermogenesis-related genes such as UCP1, PRDM16, PGC1*α*, nuclear respiratory factor 1, and mitochondrial transcription factor A [117, 120]. Moreover, by increasing PGC1*α* expression, fenofibrate increases irisin levels and therefore UCP1 expression [120]. Interestingly, through the same mechanism, fenofibrate induces the browning of WAT adipocytes in SCAT [120]. Fenofibrate decreases uptake of FA in AT due to the reduction of LPL activity [121] and the increase of HSL activity [122], respectively, decreasing lipogenesis and increasing lipolysis [121].

On the other hand, fenofibrate was also found to increase adiposity in epididymal, liver, and kidney AT in an insulin-resistant and hypertriglyceridemic rat model [123]. In humans, fenofibrate treatment has been shown to increase TG synthesis in the liver, leading to hepatic steatosis [112].

Most of the evidence suggest that fibrates, by decreasing body weight [114, 117–120, 124, 125], reduce plasma leptin concentration and increase caloric intake [114].

#### 4.2.2. Effects on Inflammation

Fenofibrate enhances adiponectin [67, 126] (HMW form in hypertriglyceridemic patients [126]) and vaspin expression and secretion [124] and in high concentrations diminishes MCP1 [127] and TNF*α* secretion [67, 125, 127, 128]. In a coculture of 3T3-L1 adipocytes and RAW264 macrophages, the TNF*α* lowering effect induced by fenofibrate was related to the inhibition of the NF*κ*B pathway [127], without changes in macrophage infiltration and lipolysis [127].

In an obesogenic environment, there is a reduction of VAT AdipoR1 and 2 protein expression [129] supporting that VAT is more prone to inflammatory processes. Fenofibrate upregulates AdipoR2 expression in 3T3-L1 adipocytes [129] and in combination with statins lowers other proinflammatory adipokines [67]. Furthermore, bezafibrate also downregulates PPARD and TNF*α* expression, while upregulating FABP4 [113] and adiponectin expression (partially through PPAR*α*, enhancing the PPRE site located in adiponectin promoter) in adipocytes [130, 131]. In contrast with other species, in human adipocytes, fenofibrate does not seem to regulate visfatin [126].

In TNF*α*-stimulated adipocytes, fenofibrate upregulates the expression of SIRT1 through the activation of the AMPK pathway [132], thus inducing NFkBp65 deacetylation and the expression of adipocyte cluster of differentiation 40 (CD40) (a costimulatory protein present in antigen-presenting cells, essential for their activation in inflammatory pathways), attenuating the obesity-related low-grade chronic inflammation state [132].

Bezafibrate lowers 11B-HSD1 mRNA expression in AT and the liver and its activity in adipocytes [130].

Aldehyde oxidase 1 (AOX1) is an enzyme responsible for drug catabolism and activation [133] producing ROS. Fenofibrate, partially by PPAR*α* stimulation, reduces protein AOX1 expression [133] leading to both antioxidant and anti-inflammatory effects [133].

#### 4.2.3. Effects on Atherogenesis

Fenofibrate increases the uptake and degradation of oxLDL by adipocytes [134], and the downregulation of PPAR*γ* and upregulation of SRB1 expression in AT are the mechanisms known to be involved in these effects [134]. Thus globally, fibrates seem to reduce adiposity and atherogenesis, despite the underlying molecular mechanisms that remain to be elucidated [119, 128, 134].

#### 4.2.4. Effects on Insulin Sensitivity

Fenofibrate can improve insulin sensitivity even in insulin-resistant models. This drug increases basal and insulin-stimulated glucose uptake by adipocytes [119] and lowers plasma FFA, TG, insulin, and glucose concentrations [120, 125]. Besides lowering TNF*α* expression, fenofibrate also decreases leptin expression [120, 125] improving insulin secretion in the postprandial period [120]. Fibrates upregulate phosphoenolpyruvate carboxykinase expression in adipocytes [135] retaining the FA output from AT to the bloodstream [123, 135].

#### 4.2.5. Effects on Adipogenesis

Through direct binding to PPAR*α*, fibrates induce adipogenesis [115], increasing the activity of enzymes involved in FA synthesis and leading to lipid accumulation in small and numerous droplets in adipocytes [116, 122, 136]. In orbital fibroblasts, fibrates also upregulate mRNA and protein expression of the nonhistone chromosomal high-mobility group AT-hook 2, leptin, and functional TSH receptor inducing preadipocyte differentiation [137].

Furthermore, fibrates reduce LPL activity, which suggests that they could rise the concentration of serum lipoproteins serving as substrates for TG storage in adipocytes [122].

### 4.3. Niacin (Nicotinic Acid)

Niacin is one of the most effective agents currently available for increasing HDL levels [138]. It acts as an inhibiting hepatocyte HDL-apoA-I holoparticle receptor responsible for HDL catabolism. Moreover, studies have demonstrated that niacin increases PPAR expression, through macrophage ABCA1, which affects reverse cholesterol transport [139]. Niacin also affects the remaining lipid profile, decreasing total cholesterol, LDL, TG, and lipoprotein (a) levels [138, 140]. Niacin is able to decrease TG synthesis and its availability for VLDL assembly, resulting in increased posttranslational intrahepatic apo-B degradation, thus decreasing plasma TG and liver secretion of apo-B-containing lipoproteins, including VLDL and LDL particles [139]. In respect to deleterious effects, at high dosages, niacin increases uric acid levels and can aggravate glucose levels [53]. Niacin is used in high doses in refractory dyslipidemia treatment, despite its limited use due to poor tolerability [141, 142].

#### 4.3.1. Effects on Adipocyte Metabolic Functions

Niacin, through HM74a receptor (coupled to Gi/o proteins [143]), reduces basal [140, 144, 145] and noradrenaline- (NA-) induced release of plasma FFA [146] and inhibits lipolysis [144–146].

Chronic treatment with niacin was shown to decrease plasma FFA levels, despite a rebound effect that can later occur [147]. The prolonged treatment with niacin enhances B-AR responsivity via postreceptor signaling modifications [147, 148]. Moreover, niacin also decreases the expression of genes involved in TG synthesis and FFA re-esterification [147]. Additionally, a decrease in perilipin and adipose phospholipase A2 protein expression could also contribute to FFA rebound [147].

Long-term niacin treatment also increases n-3-polyunsaturated fatty acid (PUFA) synthesis in AT, but not in the liver [149], thus suggesting that the main source of n-3 PUFAs is AT through lipolysis [149]. In this regard, niacin leads to upregulation of unsaturated FA biosynthesis genes (namely, Elovl6, Elovl5, and Tecr) in hyperlipidemic mice, thus increasing elongation, but not desaturation of FA [149]. Although prolonged niacin treatment enhances plasma n-3 PUFA levels, it does not significantly alter arachidonic acid-derived proinflammatory oxylipins [149]. This effect on n-3 PUFAs also contributes to CV protection since these fatty acids directly compete with n-6 PUFAs [149].

#### 4.3.2. Effects on Inflammation

Niacin reduces MCP1, RANTES, fractalkine (involved in macrophage and T cell inflammatory recruitment) gene, and protein expression, thus inhibiting macrophage chemotaxis [150]. It also decreases TNF*α*-induced iNOS gene expression lowering ROS synthesis [150]. Niacin increases adiponectin gene expression in adipocytes without affecting its secretion [150]. Binding of niacin to HM74a receptor increases adiponectin secretion in adipocytes from MS patients [145]. In these patients, acute treatment with niacin decreases plasma NEFA concentrations (without affecting both resistin and leptin concentrations) [145]. However, others described that chronic niacin treatment increases leptin levels even without changing other adipokines [151].

#### 4.3.3. Effects on Atherogenesis

Niacin enhances the cholesterol efflux rate in adipocytes through, at least partly, PPAR*γ* activation and consequently LXR*α* (liver X receptor *α*, an essential transcriptional factor for metabolism and transport of cholesterol in peripheral tissues) and ABCA1 transporter expression [140, 152]. Through this mechanism, niacin is able to increase HDL-induced cholesterol efflux from adipocytes and plasma HDL levels [140, 152]. The mechanism involved in the overexpression of these factors is unclear, although it has been pointed out as a role for HM74a as the initial trigger [140].

Niacin stimulates PPAR*γ* expression and activity increasing anti-inflammatory prostaglandin synthesis and secretion by macrophages [153]. Nevertheless, prolonged treatment with niacin seems not to modify endothelial function and inflammatory activity in MS patients [151].

#### 4.3.4. Effects on Insulin Sensitivity

Prolonged treatment with niacin seems to induce insulin resistance [148, 151]. In fact, in dyslipidemic mouse models, niacin downregulates genes involved in insulin (such as INSR and PDE3B) and B-adrenergic (such as B-1,2,3-AR) signaling pathways [148], whereas prolonged treatment enhances B-AR responsivity [147, 148]. The authors suggest that the duration needed to increase adiponectin levels could be counterbalanced by other adverse effects, such as the rebound increase in plasma FFA [151].

#### 4.3.5. Effects on Adipogenesis

Niacin stimulates adipogenesis (enhancing PPAR*γ*, FABP4, adiponectin, and leptin expression) in 3T3-L1 cells, while it suppresses C/EBPB and thereby cyclooxygenase-2 expression, responsible for PGF2a (antiadipogenic factor) decrease in adipocytes [154].

### 4.4. Ezetimibe

Ezetimibe acts by inhibiting intestinal cholesterol absorption (through Niemann-Pick C1-Like 1 (NPC1L1) transporter) and by decreasing its delivery to the liver, leading to upregulation of hepatic LDL receptors [53]. This drug can reduce LDL levels by 10%–18% or 34%–61%, respectively, used as monotherapy or in combination with statins [53]. Ezetimibe is also able to reduce Apo-B levels (by 11%–16%) [53].

#### 4.4.1. Effects on Adipocyte Metabolic Functions

Ezetimibe decreases fat visceral accumulation, without affecting total body weight [155] and improves hepatic steatosis [155].

#### 4.4.2. Effects on Inflammation

Ezetimibe was shown to reduce visfatin, while increasing adiponectin plasma levels [155, 156]. The combination of ezetimibe-simvastatin treatment for 30 days was able to partially revert AT dysfunction and decrease systemic inflammation, independently of the lipid-lowering effect [157]. The same combination also seems to decrease leptin, visfatin, and TNF*α* and increase adiponectin levels [157].

#### 4.4.3. Effects on Insulin Sensitivity

Ezetimibe is able to improve insulin resistance, particularly in patients with MS [155] and seems to be more potent in insulin-resistant patients [156].

The effects of drugs used in dyslipidemia on AT functions are summarized in Table 1 and Figures 1 and 2.

## 5. Drugs Used in Obesity

### 5.1. Orlistat

Orlistat inhibits gastric and pancreatic lipases [158], enzymes that play a pivotal role in the digestion of dietary fat. Thus, orlistat by impairing fat intestinal absorption leads to body weight reduction (weight loss of 3% (https://www.gene.com/download/pdf/xenical_prescribing.pdf, accessed on 15th February 2017)), improves glucose intolerance, and ameliorates lipid parameters (total cholesterol and LDL) [159, 160]. Interestingly, orlistat can reverse liver steatosis but not adipocyte hypertrophy [159].

#### 5.1.1. Effects on Adipocyte Metabolic Functions

Orlistat partially inhibits lipolysis in adipocytes by suppressing AMPK activation and decreasing the AMP/ATP ratio induced by forskolin, isoproterenol, and IBMX (agents able to increase cAMP levels), without altering PKA activity and cAMP levels [161]. On the other hand, others reported that orlistat has a lipolytic effect, inducing TG degradation in AT and the liver [160].

#### 5.1.2. Effects on Inflammation

Orlistat combined with a hypocaloric diet was able to produce a marked reduction in plasma concentrations of leptin, CRP, IL-6, TNF*α*, and resistin, while increasing adiponectin levels [162, 163]. Globally, the available literature points to a role of orlistat in improving obesity-related AT dysfunction [162, 163].

### 5.2. Anorexiants/Central Nervous System Stimulants

#### 5.2.1. Sibutramine

Sibutramine is an inhibitor of NA and 5-hydroxytryptamine (5-HT) neuronal reuptake, no longer used due to deleterious cardiovascular side effects. This drug induces a weight loss of 5%, decreases waist circumference, serum TG, and CRP, while increasing serum HDL levels and insulin sensitivity [164]. Globally, this drug exhibits an anti-inflammatory role, as it lowers leptin and resistin levels and increases adiponectin [164].

#### 5.2.2. Diethylpropion

Diethylpropion, a sympathomimetic amine similar to amphetamine, is a prodrug metabolized to 2-ethylamino-1-phenyl-propan-1-one and N, N-diethylnorephedrine metabolites [165], the latter being responsible for its effects. This metabolite acts as substrate for NA transporter, inhibiting NA reuptake, while stimulating its release [165]. Therefore, higher NA concentrations in the brain could justify the anorexiant effect and the side effects common to amphetamine use [165]. It also acts as a reuptake inhibitor of both DA and 5-HT transport [165].

#### 5.2.3. Phentermine and Lorcaserin

Phentermine is a sympathomimetic amine similar to amphetamine but with residual additive potential. It also acts as DA receptor agonist and as NA receptor partial agonist or antagonist [166], while lorcaserin [167, 168] is a 5-HT 2c receptor agonist. Both drugs decrease food intake and increase satiety and cause weight losses of [166–168], respectively, 5% (https://www.accessdata.fda.gov/drugsatfda_docs/label/2012/085128s065lbl.pdf, accessed on 15th February 2017) and 8% (https://www.belviq.com/-/media/Files/BelviqConsolidation/PDF/belviqxr_prescribing_information-pdf.pdf?la=en, accessed on 15th February 2017).

### 5.3. Antidepressants

#### 5.3.1. Naltrexone and Bupropion

Naltrexone is an antagonist of opioid receptors in pro-opiomelanocortin (POMCs) neurons and bupropion, a NA and DA reuptake inhibitor. The combined treatment with these drugs decreases food intake, body weight (weight loss exceeding 8% of baseline (http://general.takedapharm.com/content/file.aspx?filetypecode=CONTRAVEPI&CountryCode=US&LanguageCode=EN&cacheRandomizer=bc8d4bba-8158-44f2-92b3-1e1ba338af0a&cacheRandomizer=5fa7daab-0bf1-44e1-8c26-f51e7f3a6c09, accessed on 15th February 2017)), and fat mass (without changing lean mass) in diet-induced obese rats [169, 170]. One of these studies also reported the decrease of VAT mass with this combination [170]. Adding amylin (a peptide coreleased with insulin by pancreatic B cells) to these drugs seems to result in better outcomes [169], due to the modulation of the melanocortin (MC) pathway (increasing the expression of MC4 receptor in hypothalamic neurons) [169].

### 5.4. Antiepileptics

#### 5.4.1. Topiramate

Topiramate is an antiepileptic drug that acts as antagonist of AMPA (a-amino-3-hydroxy-5-methyl-4-isoxazolepropionic acid) receptors and positively modulates *γ*-aminobutyric acid (GABA) receptors. Topiramate significantly induces weight loss and decreases glycemia, insulinemia, insulin resistance, and TG, while increases adiponectin plasma levels in diet-induced obesity rats [171]. Topiramate does not affect arterial pressure or anxiety, by not fully understood mechanisms. Nonetheless, inhibition of food ingestion, downregulation of leptin expression, and upregulation of UCP-2 and 3 expression in WAT and BAT seem to be involved [172].

### 5.5. Liraglutide

Liraglutide is a glucagon-like peptide 1 receptor agonist (GLP-1RA), firstly approved as antidiabetic drug and, more recently, in higher doses as antiobesity drug, providing a weight loss of 9% (http://www.novo-pi.com/saxenda.pdf accessed on 15th February 2017). GLP-1 is an endogenous incretin secreted by L cells in the distal intestine [173, 174]. Liraglutide, by increasing GLP-1 levels, reduces food ingestion and appetite [175–180] and modifies food preferences, namely, improving eating e and decreasing emotional eating, which increases weight loss [175, 177]. Liraglutide can also slow gastric emptying [178], a mechanism that helps to reduce food intake. Moreover, this drug can have broader effects on metabolism, as GLP-1 was described as having antiadipogenic, antilipogenic, and prolipolytic effects in human mature adipocytes [181] and as activating GLP-1R in the central nervous system (CNS) leading to an increase in BAT activity and energy expenditure [180].

#### 5.5.1. Effects on Adipocyte Metabolic Functions

Liraglutide reduces total fat mass and fat thickness from different depots [175, 182–188]. Noteworthy, liraglutide is also capable of changing regional distribution of fat depots [175, 176, 178, 182–187], acting mainly by decreasing VAT [175–178, 182–187], a result not confirmed by another study describing a preferential effect on SCAT [184].

The liraglutide-induced weight loss seems to increase NP concentrations [182], which induce lipid oxidation [182, 188]. The ANP and BNP increase is higher in patients losing more than 5% of weight and significantly correlate with liraglutide effects on body composition [182]. In addition, lipid storage reduction in WAT decreases lipogenesis [176]. These effects seem to be driven by downregulation of Akt and PI3K pathways and upregulation of AMPK and ACC genes [176]. Furthermore, liraglutide was shown to increase energy expenditure, by inducing WAT and BAT browning and increasing thermogenesis [180, 182, 188]. This browning effect was also shown to be driven by an increase in NP through MAPK pathway stimulation [182]. Nevertheless, the magnitude of BAT activity increase is modest and does not justify the extent of liraglutide effect in weight loss [180]. Liraglutide, through stimulation of CNS GLP-1R on ventromedial hypothalamic nuclei and modulation of AMPK pathway, was shown to decrease body weight [188], independently of the 5-HT2CR and MC4R pathways [179].

#### 5.5.2. Effects on Inflammation

Liraglutide has been described to regulate adipokine secretion in opposite directions. In T2DM patients, this drug decreases total adiponectin levels while increasing pentraxin 3, a marker of inflammatory CVD, and proinsulin levels [184]. This latter effect demonstrates a beneficial role on pancreatic B cells [184]. Conversely, in obese patients, liraglutide increases adiponectin expression and inhibits glucose uptake in adipocyte stem cells [189] and, in human adipocytes, decreases TNF*α* and adiponectin expression [181].

#### 5.5.3. Effects on Atherogenesis

Liraglutide decreases CRP levels and soluble ICAM-1 [175] seeming therefore to have pleiotropic and antiatherosclerotic effects [175].

#### 5.5.4. Effects on Obesity-Related Cardiovascular Comorbidities

GLP-1R is more expressed in adipocytes from VAT of obese T2DM patients, comparing to lean patients [181]. Liraglutide can improve insulin sensitivity, even in insulin-resistant models. Omentin (an adipokine mainly produced by VAT), through Akt/PKb signaling pathway stimulation [190, 191], increases glucose transport induced by insulin hence improving insulin sensitivity and glucose metabolism [192]. The omentin plasma levels are decreased in T2DM and liraglutide can increase its levels [192]. Liraglutide increases ZAG (zinc *α*2 glycoprotein), a protein involved in multiple effects such as body weight control and lipolysis, and adiponectin plasma levels. Moreover, liraglutide improves insulin secretion [178, 182, 185, 192] and consequently glucose uptake in peripheral tissues [179]. This drug also increases PPAR*γ* activity and therefore liver production of fibroblast growth factor-21 (FGF21) that leads to an increase in FGF21 plasma levels [179]. In obese and T2DM patients, FGF21 mRNA expression and plasma levels are elevated, a compensatory mechanism to decrease insulin resistance. The resultant decrease of FGF receptor (FGFR) supports FGF21 resistance in these conditions [185]. Furthermore, liraglutide also upregulates the expression of FGFR3 and B-Klotho (necessary to the binding of FGF21 to its receptor) in AT, while in the liver upregulates FGFR1-3, B-klotho, and phospho-FGFR1 expression [185]. Since FGF21 is an important regulator of insulin effects on glucose and lipid metabolism, liraglutide could contribute to improve insulin action [179, 185].

Conversely to other studies, showing no effect on fat liver parameters [183], liraglutide has been shown to decrease hepatic fat, including in obese and/or T2DM patients [184, 186, 193]. The decrease in intrahepatic lipids does not correlate with changes in weight, abdominal fat, VAT, SCAT, or adiponectin levels, but rather with a decrease in HbA1c [193]. Authors anticipated that this effect is due to an increase in glucose tolerance, thus reducing hyperinsulinemia [193] followed by a decrease in lipogenesis rate and an increase in FA oxidation. Moreover, treatment with liraglutide improves systolic blood pressure and lipid profile, decreasing plasma total cholesterol and TG while increasing HDL levels [175, 178, 182, 185, 186].

#### 5.5.5. Effects on Adipogenesis

GLP-1 and GLP-1RA are able to regulate preadipocyte differentiation, even though they act differently according to adipocyte origin or differentiation stage.

Liraglutide stimulates the early phase of adipogenesis in 3T3-L1 cells by inducing the expression of PPAR*γ*, C/EBPB and d, and GLP-1R, a target gene of PPAR*γ* [194]. It modulates both the survival and proliferation pathways, mainly ERK1/2, PKCB, and Akt [194]. In contrast, liraglutide inhibits both proliferation and differentiation of ASCs obtained from obese patients by binding directly to GLP-1R [189]. GLP-1RA decreases the expression of adipogenesis- and lipogenesis-related genes, while increasing the expression of the lipolytic ones [181]. Unlike 3T3-L1 cells, in human adipocytes, GLP-1 antiadipogenic effect is driven through inactivation of the AC/cAMP pathway [181]. The effects of drugs used in obesity on AT are summarized in Table 2 and Figures 1 and 2.

## 6. Conclusion

AT is a complex organ with marked effects on whole-body physiology. AT dysregulation, rather than the amount of fat mass, seems to be a key factor in the pathophysiology of obesity and related morbidities. Despite the increase in the number of drugs available to treat these conditions, dyslipidemia and obesity prevalence still remains rising. AT dysregulation is a main feature present in both dyslipidemia and obesity. The clinical outcomes of AT modulation by these drugs, as well as differences between them in the modulation of pathways involved in metabolism, inflammation, atherogenesis, insulin sensitivity, and adipogenesis, were identified. Whether solutions to these issues will be found in further adjustments and combinations between drugs already in use or necessarily in new advances in pharmacology is not known. To better understand the impact of drugs used in dyslipidemia and obesity on AT function not only is challenging for physicians but could also be the next step to tackle CVD.

## Figures and Tables

**Table 1:** Drugs used in dyslipidemia: classical effects, effects on adipose tissue, and weight.

Drugs used in dyslipidemia	Classical mechanism of action	Adipose tissue effects									Weight
		AT mass/AT depots	Glucose metabolism/insulin sensitivity	Lipid metabolism	Adipokine expression		Antiatherogenic	Adipogenesis	Browning effect	Anti-inflammatory	
					↑	↓					
Statins	⊝ HMG -CoA reductase enzyme	↓ EAT [63]	⊝ caveolae dysfunction [89]; ⊝ GLUT4 expression and translocation [95]; NLRP3 inflammasome activation [195]; ⊕ ↑ SIRT1 and PGC-1*α* leading to PPAR*γ* and GLUT4 [97–99]; modulation of adipokine expression [83, 97]	⊕ lipolysis [62] ↓ lipid accumulation (⊕ LPL) [64, 65] ⊕ lipogenesis and ↑ lipid accumulation [196]	Adiponectin [62, 67, 68, 73, 76, 80]	Leptin [67–71]; resistin [67, 72, 73]; IL-6 [67, 74–78]; PAI-1 [79–81]; MCP-1 [77, 78, 80, 82, 83]; visfatin [71] and TNF*α* [67, 68, 71, 77, 82, 83]	⊕ PPAR*γ* and SRBI expression (adipocyte uptake of oxLDL) [88, 90]; *vide adipokine expression modulation*	⊝ [66, 101–103] ⊕ *in vivo* [107, 108]		⊝ ER stress [82]; ⊕ iNOS expression [75, 85]	— [98, 99]
Fibrates	PPAR*α* agonists							⊕ [115, 122]			↓ [114, 117–120, 124, 125]
Bezafibrate	Nonselective			⊕ FA oxidation [113–115] ⊝ lipogenesis [113]	Adiponectin [130, 131]	TNF*α* [130, 131]			⊕ UCP-1, 2, and 3 expression [113, 114]		
Gemfibrozil	Selective			⊕ lipogenesis [116]							
Fenofibrate	Selective	↓ VAT [125]	⊕ [119, 120, 125]	⊕ FA oxidation [118, 119] ⊝ lipogenesis [121]	Adiponectin [67, 126]; vaspin [124]	MCP1 [127]; TNF*α* [67, 125, 127, 128]; leptin [120, 125]	⊕ oxLDL uptake [134] ⊕ CD36 expression [134]		⊕ [117, 120]	⊝ CD40 expression (AMPK pathway) [132] ⊝ AOX1 expression [133]	↓ [117–120, 125]
Ezetimibe	⊝ NPC1L1 transporter	↓ VAT [155]	⊕ [155]		Adiponectin [155]	Visfatin [156]					— [155]
Niacin	⊝ HDL-apoA-I holoparticle receptor in hepatocytes		⊝ [148, 151]	⊕ lipolysis [144–146] ⊝ lipogenesis	Adiponectin [145]; leptin (chronic treatment) [151]	MCP1, RANTES, fractalkine [150]	↑ n-3 PUFAs and its metabolites [149]; ⊕ HDL-induced cholesterol efflux from adipocytes; ↑ HDL levels [140, 152]	⊕ [154]		*Vide adipokine effects*	

⊕: stimulates; ⊝: inhibits; —: without effect; AT: adipose tissue; TG: triglycerides; HMG-CoA: 3-hydroxy-3-methyl-glutaryl-coenzyme A; GLUT4: glucose transporter type 4; NLRP3: NOD-like receptor family, pyrin domain containing 3; SIRT1: sirtuin 1; PPARs: peroxisome proliferator-activated receptors; PGC-1*α*: peroxisome proliferator-activated receptor *γ* coactivator 1 alpha; LPL: lipoprotein lipase; TNF*α*: tumour necrosis factor *α*; IL: interleukin; CCL2 or MCP-1: CC-chemokine ligand 2; PAI-1: plasminogen activator inhibitor type 1; SRB1: scavenger receptor 1; oxLDL: oxidized LDL; CD36/FAT: fatty acid translocase; ER: endoplasmic reticulum; iNOS: inducible nitric oxide synthase; UCP: uncoupling protein; NPC1L1: Niemann-Pick C1-Like 1; VAT: visceral AT; FA: fatty acid; AMPK: adenosine monophosphate-activated protein kinase; AOX1: aldehyde oxidase 1; CD40: cluster of differentiation 40; sICAM-1: soluble intracellular adhesion molecule-1; HDLs: high-density lipoproteins; PUFAs: n-3 polyunsaturated fatty acids.

**Table 2:** Drugs used in obesity: classical effects, effects on adipose tissue, and weight.

Drugs used in obesity	Classical mechanism of action	Adipose tissue effects									Appetite regulation
		AT mass/AT depots	Glucose metabolism/insulin sensitivity	Lipid metabolism	Adipocytokine expression		Antiatherogenic	Adipogenesis	Browning effect	Anti-inflammatory	
					↑	↓					
Orlistat	Reversible inhibitor of gastric and pancreatic lipase		⊕ [159, 160]	⊕ lipolysis [160]		Leptin [160]					
Sibutramine	Sympathomimetic amine; inhibition of NA and 5-HT reuptake			↓ TG [164] ↑ HDL [164]	Adiponectin [164]	Leptin and resistin [164]	↓ CRP [164]				
Diethylpropion	Sympathomimetic amine; inhibition of NA, 5-HT; DA reuptake										⊝ [165]
Phentermine	Sympathomimetic amine; noradrenergic modulation; DA receptor agonist										⊝ and promotes saciety [166]
Lorcaserin	5-HT2c receptor agonist										⊝ and promotes saciety [167, 168]
Naltrexone and bupropion	Antagonist of opioid receptors in POMC neurons and inhibitor of reuptake of NA and DA	↓, mainly VAT [170]									
Topiramate	Antagonist of AMPA receptors and stimulation of GABA receptors		⊕ [171]		Adiponectin [171]	Leptin [172]			⊕ UCP 2/3 expression [172]		⊝ [172]
Liraglutide	GLP-1R agonist	↓ [175, 182–188]; mainly VAT [175–178, 182–187]	⊕ [178, 182, 185, 192]	⊕ FA oxidation [182, 188]; ⊝ lipogenesis [176]	Adiponectin (in dysfunctional adipocyte) [189, 197]; omentin [192]	TNF*α* [181]	↓ CRP and sICAM-1 levels [175]	⊕ [194]	⊕ [180, 182, 188]	*Vide adipokine regulation*	⊝ and promotes saciety and improves eating behaviour [175–180]

⊕: stimulates; ⊝: inhibits; AT: adipose tissue; TG: triglycerides; HDLs: high-density lipoproteins; CRP: C-reactive protein; NA: noradrenaline; 5-HT: 5-hydroxytryptamine; DA: dopamine; POMC neurons: pro-opiomelanocortin neurons; VAT: visceral AT; AMPA receptors: a-amino-3-hydroxy-5-methyl-4-isoxazolepropionic acid receptors; GABA receptors: *γ*-aminobutyric acid receptors; FA: fatty acid; TNF*α*: tumour necrosis factor *α*; GLP-1R: glucagon-like peptide 1 receptor; sICAM-1: soluble intracellular adhesion molecule-1.
